# Artificial Intelligence in Elite Sports—A Narrative Review of Success Stories and Challenges

**DOI:** 10.3389/fspor.2022.861466

**Published:** 2022-07-11

**Authors:** Fabian Hammes, Alexander Hagg, Alexander Asteroth, Daniel Link

**Affiliations:** ^1^Chair of Performance Analysis and Sports Informatics, Department of Sport and Health Science, Technical University of Munich, Munich, Germany; ^2^Computer Science, Institute of Technology, Resource and Energy-Efficient Engineering, Bonn-Rhein-Sieg University of Applied Sciences, Sankt Augustin, Germany; ^3^Munich Data Science Institute, Technical University of Munich, Munich, Germany

**Keywords:** artificial intelligence, elite sports, SMPA loop, explainable AI, AI usage in sports

## Abstract

This paper explores the role of artificial intelligence (AI) in elite sports. We approach the topic from two perspectives. Firstly, we provide a literature based overview of AI success stories in areas other than sports. We identified multiple approaches in the area of Machine Perception, Machine Learning and Modeling, Planning and Optimization as well as Interaction and Intervention, holding a potential for improving training and competition. Secondly, we discover the present status of AI use in elite sports. Therefore, in addition to another literature review, we interviewed leading sports scientist, which are closely connected to the main national service institute for elite sports in their countries. The analysis of this literature review and the interviews show that the most activity is carried out in the methodical categories of signal and image processing. However, projects in the field of modeling & planning have become increasingly popular within the last years. Based on these two perspectives, we extract deficits, issues and opportunities and summarize them in six key challenges faced by the sports analytics community. These challenges include data collection, controllability of an AI by the practitioners and explainability of AI results.

## Introduction

The idea of *artificial intelligence (AI)*, machine behavior that would be considered intelligent if exhibited by humans, is as old as human built machines. More recently, the field of AI emerged as a subfield of computer science in the 1950's. Since then the research field has gone through several ups and downs – the so-called AI winters in the mid 70's and late 80's. Since the end of the 90's, a continuous upwards trend is discernible. This positive trend is due to multiple factors. The first factor is the invention of machine learning (ML) and deep learning (DL) methods. The second influence is the substantial increase in computational power combined with an increasing digitization in all areas. This combination has enabled the processing of huge amounts of data (“Big Data”) during the last two decades. McCorduck (2004) gives a historical overview of the developments in this area. Today, AI has penetrated the everyday life of people in ways that did not seem possible 10 or 15 years ago. Topics like autonomous driving (Grigorescu et al., 2020) or virtual assistants, like Amazon Alexa, Siri or Cortana (Maedche et al., 2019) are well-known examples of current day AI. Another famous, recent example is the victory of the AI system AlphaGo against the world's best Go-Player in 2016 (Silver et al., 2016). These developments emphasize that the rate of AI development is currently reaching new heights, taking many people by surprise (Wang et al., 2016). These are only some examples of what AI has achieved within the last decade.

On a smaller scale, AI has also affected the world of sports [overviews by Beal et al. (2019), Claudino et al. (2019), Araújo et al. (2021)]. One famous example can be found in the so-called “Moneyball years” of the baseball team Oakland Athletics at the start of the 2000's. During these years, a new approach to evaluate statistics with machine learning (ML) led to a massive change in their behavior on the transfer market. This is often referred to as the birth of AI in elite sports (Lewis, 2004). The team's success showed that their approach worked and a growing number of teams in various kinds of sports put more focus on using ML for handling data. However, not only is the player recruitment affected, there are many other areas influenced by ML nowadays. Referee support – e.g., via hawk-eye in tennis (Owens, 2003) or goal-line technology in football (FIFA, 2014), tactical behavior (Le et al., 2017), automated training planning (Skerik et al., 2018) or rehabilitation (Claudino et al., 2019) are just some examples where AI techniques have benefited sports. Support services of sport events, like broadcasting, spectator experience or the betting industry often use techniques of AI (Martinez-Arastey, 2021). Today, a worldwide research community has been established to treat the question of AI in sports, for example through the MIT Sloan Sports Analytics Conference and IACSS Conference (International Association of Computer Science in Sport).

One of the first questions that has to be clarified, when exploring the role of AI in sport, is the definition of AI. The sport (scientific) community uses the term AI as a kind of metaphor for an advanced, data driven technology, without having a concrete definition or framework in mind. For the purpose of this paper, we define AI based on the Sense-Model-Plan-Act loop (SMPA). Although there are other concepts of AI (e.g. reactive and subsumption), this model provides a good structure criterion for organizing this paper and will serve as a structuring element for it as well (Brooks, 1986).

**Figure 1:**
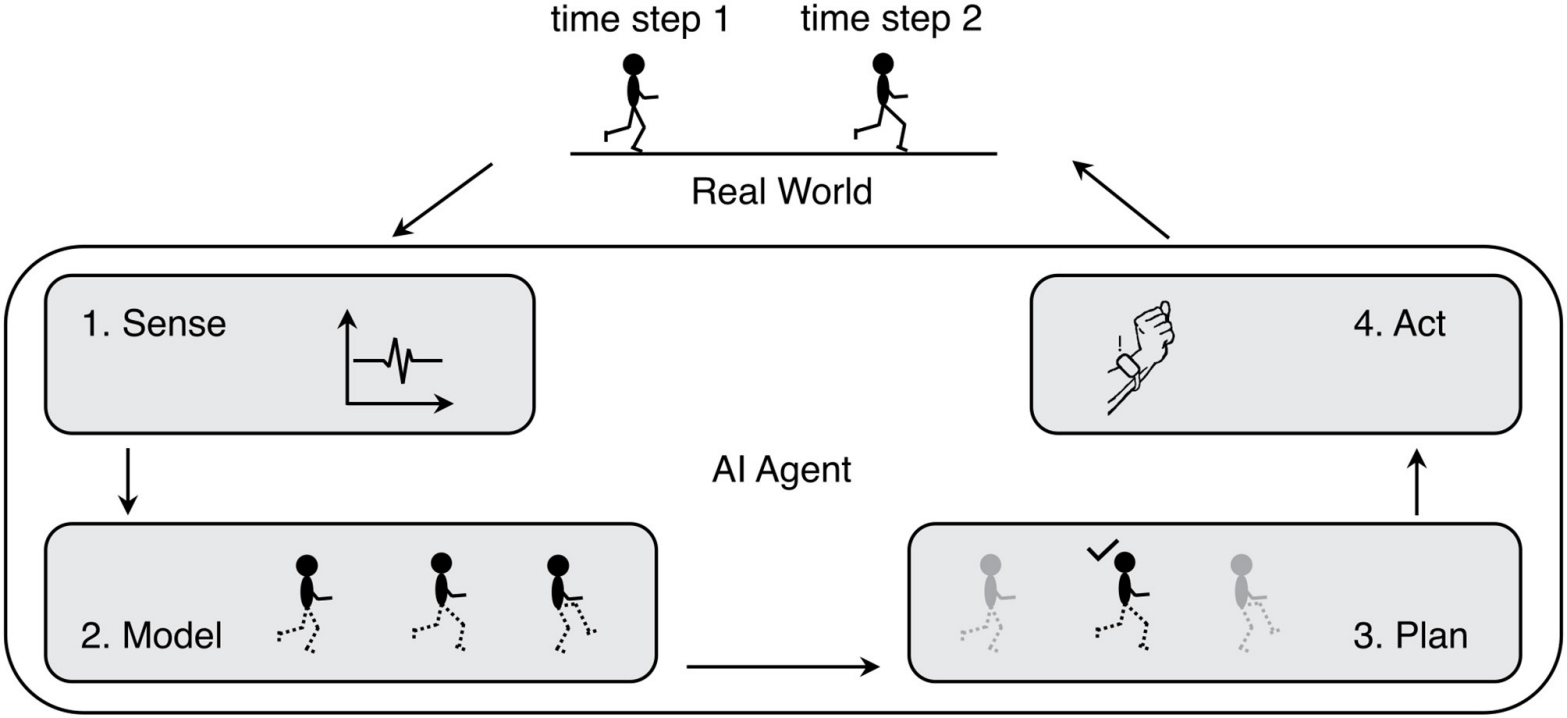
Sense-Model-Plan-Act loop (SMPA).

The SMPA concept defines AI as a loop that perceives and acts upon the world by modeling its perception, creating a deliberate plan based on that model and formulating an action, which is then exerted on the world. The parts of this definition are illustrated by Figure 1. A machine demonstrating intelligent behavior does so by interacting with its environment. Therefore, such machines are usually referred to as intelligent agents. The process can be seen as a loop of four actions: An agent receives sensory information from its environment (1. Sense). In sports this information can refer to e.g., heart rate data, image data, or entire video data streams. Then, the agent builds a *predictive* model based on these perceptions (2. Model). Nowadays, this model is usually statistical, data driven and employs machine learning methods. The model can predict the quality of result of e.g., a workout scheme, the impact of a strategy in team sports, or other factors that determine the ability, quality, and robustness of plans executed in the environment. The predictive model can then be used to e.g., search and optimize action planning such as a match plan or a training schedule (3. Plan). The resulting strategy is carried out, e.g., by means of feedback to the user, thus acting on, or interacting with the environment (4. Act). In sports this could be a device, which gives real time feedback to the user (e.g., pace control by vibration) or proposes promising strategies by using visualizations. The SMPA shows that AI is much more than data mining or machine learning. It involves multiple steps that all, in themselves, are fields of research on their own. All four steps form a loop that is connected via the real world environment providing feedback and thus allowing self-adaptation – the most important prerequisite for intelligent behavior.

Against this background, our paper firstly identifies success stories of AI in fields outside the sports domain. The focus here lies on technologies, which can be linked to possible future use in elite sports. Secondly, we look at the current use of AI in elite sports. This is done based on a literature review as well as on interviews with international experts. These interviews addressed three main themes. They discussed the use of AI in the national supporting institutes for elite sports in the respective countries. Moreover, the requirements for success applications were highlighted, and the limitations of the concept were also investigated. Thirdly, the paper puts these two parts together and asks which success stories of AI are not utilized in elite sports so far. The result is a list of challenges for the scientific community to uncover the potential application of AI in elite sports. They might be useful as an orientation for scientists working in this area as well as for the national funding agencies for sports science in steering research activities.

## Success Stories of AI Outside the Sports Domain

### Method

In order to identify the most successful strands of AI applications, we performed an extensive literature review. A naive attempt to do a Google Scholar search on the keyword “machine learning” from 2000-2020 yields nearly two million matches. This makes it impossible to provide a comprehensive and systematic overview of the recent development in the field of AI in the way it is defined by PRISMA (Page et al., 2021) or similar approaches. Therefore, we choose a narrative review and followed the methodological guidelines proposed by Ferrari (2015). One limitation of this is that our selection of literature may be biased; nevertheless, this allows identify many key areas in which AI was successfully applied, which is our primary scope. We narrowed down our search using the following strategy: we searched for the most cited survey papers for all four parts of the SMPA loop of AI. Using these as a starting point, we focused on the most important developments of the last 10 years. Special focus was put on success stories in which we see a potential transfer into sports. Finally, factors that led to success in each case were identified. These factors were then compared with conditions that we find in sports applications.

### Results

#### Machine Perception

Image recognition and computer vision techniques are widely used for action recognition. Many basic applications, for example in robotics, video surveillance, and human-computer interaction, are based on these techniques. An overview and categorization of the approaches focuses on those techniques that aim to classify whole-body movements, such as kicking and punching, from an input stream of visual data (Weinland et al., 2011). Features are extracted from video footage, which can then be used to segment, classify, and learn actions. There are some important issues that are not addressed in most of the literature: e. g. scalability of action recognition systems, dealing with unknown motion, dealing with occlusions, and scenes with multiple people.

The use of multimodal camera systems, combining for example color images and depth data, has contributed to improving motion detection. Additionally, these systems have also helped object classification in more complex environments, at least since the transfer of such solutions from the gaming industry to robotics (Lun and Zhao, 2015; Hagg et al., 2016; Wang et al., 2018). Multimodal systems have enabled the extraction of skeletal model data from human movements. Large, multimodal training datasets are freely available (e.g., Shahroudy et al., 2016; Liu et al., 2020), but not all action sequences useful for sports are represented in the datasets.

A promising area for sports is non-visual sensor data. The data provided by the increasingly diverse range of so-called wearables (wearable computer systems) is quite similar to the data collected in elite sports. Current research is dominated by the *wristwatch-as-a-wearable*. Connecting wearable devices to broader Internet of Things (IoT) ecosystems may enable new services and interaction modalities.

Another underdeveloped research area concerns wearable devices that are used for sports rehabilitation (Mencarini et al., 2019). Even if the use of wearables in elite sports turns out to be unsuitable in some cases, due to e. g. competition rule systems, they can very well be used for training and during rehabilitation (Füller et al., 2015; Ludwig et al., 2015, 2019; Schaefer et al., 2015). However, the research contains some gaps. In fact, only 10 of the 57 papers reviewed in Mencarini et al. (2019) deal with elite athletes.

By using multiple sensor types, the robustness of perception in AI systems against e. g. noise and sensor weaknesses can be increased in many cases. Examples can be found in gesture recognition (Nishida and Nakayama, 2015), prediction of physiological stress (Parent et al., 2019), classification (Choi and Lee, 2019), detection of Parkinson (Vásquez-Correa et al., 2018), and three-dimensional navigation (Barbot et al., 2016).

#### Machine Learning and Modeling

In modern times, the greatest developmental leap in AI took place in the shape of layered models that can learn patterns based on large amounts of data (DL). The models are called “deep” due to the large number of layers within them. There are many examples of these models' successful applications in the last decade (Das and Behera, 2017; Litjens et al., 2017; Kamilaris and Prenafeta-Boldú, 2018). One particularly interesting tangential application is in medical computing. This is because both, medical computing and elite sports deal with human physiology (Litjens et al., 2017). Unsupervised training methods, i.e., the creation of methods based on non-annotated data, should be considered as well. Methods such as variational autoencoders and generative adversarial networks stand out for (pre)processing of large unlabeled data sets, and find use in applications like anomaly detection (An and Cho, 2015).

Sensor-based activity recognition attempts to recognize human activity from a variety of raw data/sensor values. Conventional methods often rely heavily on heuristic, hand-crafted feature extraction, which can hinder their generalization performance. DL-based methods have largely arrived for sensor-based activity recognition tasks (Wang et al., 2019). The authors treat the detection of activities using wearables with the aid of DL. They note that DL can overcome some weaknesses of classical methods, and greatly simplify modeling. One of the methods of doing this is by being able to inject large amounts of unqualified data into models and applying unsupervised learning before learning the qualified data. Unsupervised learning methods are used on unlabeled data to uncover structure and correlation within the data. The aforementioned article should be taken as a starting point to the possibilities of DL in combination with wearables, as there are still specific issues that would need to be addressed.

Transfer learning (Lu et al., 2015; Pan, 2016) refers to ML models that transfer learned knowledge from one domain to another. In elite sports, the transfer learning method has the potential to be pre-trained on sports that provide a lot of data and then transferred to sports that provide only limited data.

The increasing dependence on algorithms and models can lead to systematic biases and decision failures. Explainable AI is concerned with implementing transparency and traceability of black-box statistical ML and DL methods. Even though there is no generally accepted definition of Explainable AI the purpose is always that results of a used method must be interpretable for humans (Doran et al., 2017). Especially in areas where there is direct physical and mental impact on humans, it is imperative to understand the modeling techniques and models used. Apart from this, it is also necessary to demand transparency regarding automated modeling and decision-making. Explainable AI is also attracting considerable interest in medicine (London, 2019). Even though classical AI systems provided comprehensible approaches, their weakness was dealing with uncertainties in the real world. DL has been more successful but increasingly opaque. Among others, Holzinger et al. (2019) argues that there is a need to go beyond explainable AI in medicine. For explainable medical decisions, one needs causality, not just statistical correlation. Holzinger et al. (2019) give definitions of the distinction between explainability and causality, and a use case of DL interpretation and human explanation in histopathology. Other developments include the development of a framework for explainable AI systems (Preece, 2018), and a framework for assessing the explainability of AI systems (Sokol and Flach, 2020). In the latter, the authors present a taxonomy and a set of descriptors used to characterize and systematically assess explainable systems along five key dimensions: functionality, operationality, usability, security, and validation.

The use of ML should remain limited to tasks where accuracy and reliability can be empirically validated. Only if ML is the most effective alternative, evaluated with an appropriate validation, it should be used. This can be done even if the reasons for the better performance remain somewhat opaque. The robustness of systems should be tested during development by examining when their accuracy and reliability break down. This includes validation and ongoing reassessment of system performance on multiple, real-world datasets (Ribeiro et al., 2016; Rosenfeld and Richardson, 2019; Tjoa and Guan, 2020).

Robust learning, i.e., learning considering erroneous or noisy data, is used in the image domain of the medical field even for smaller data sets (Kononenko, 2001). Error and risk detection appears to have long been adopted in elite sports. A large-scale study on injury risk assessment analyzed 58 trials, using 11 AI techniques or methods in 12 team sports (Claudino et al., 2019). In total 76% of the participants were professional athletes. The most commonly used AI techniques or methods were artificial neural networks, decision tree classifiers, support vector machines, and Markov processes with good performance metrics for all of them. The results of this review suggest a widespread application of AI methods in team sports based on the number of published studies.

The majority of DL models are not well understood, so-called black box models. In contrast, statistical models are often used. These models not only to determine a prediction, but also give model confidence and an estimation of noise values in the data. Here, Gaussian process (GP) regression in particular can be identified as an appropriate modeling method (Rasmussen, 2003; Vanhatalo et al., 2013). Gaussian processes do not learn a function but a statistically normal distribution of functions in most cases. The variance of the models is interpreted as the model uncertainty (inverse confidence). The original formulation of GP was not able to handle more than about 1,000 data points. In recent years, however, methods have emerged that allow this. Thus, GP is often suitable for big data as well nowadays (van Stein et al., 2015; Liu et al., 2018).

To assist in training models, there is often the difficulty of determining the correct model configuration parameters. This requires expertise, which is not always available. To increase accessibility to machine learning, even for non-experts, there is an attempt to have this configuration done automatically (AutoML). Here, the following steps, which are to be automated, can be distinguished: data preparation and ingestion (from raw data and different formats), task recognition – e.g., binary classification, regression, clustering or ranking, feature engineering, model selection, the hyper-parameter optimization of the learning algorithm and features, pipeline selection under time, memory and complexity constraints, selection of evaluation metrics and validation procedures, problem testing, analysis of the obtained results and the generation of user interfaces and visualizations. For the benefits of AutoML, the evidence base is limited. While there are many approaches and also open frameworks, there are few real-world comparisons between AutoML and, for example, augmented learning, where configuration parameters no longer need to be predetermined. What is certain, however, is that machine learning accessibility is a double-edged sword. If non-experts can gain access but cannot assess whether a model has been trained correctly, the attempt to integrate AI into sports may fail because the models might not prove themselves.

#### Planning and Optimization

Learning dynamical models that are accurate enough for planning is a long-standing challenge, especially in image-based domains. The Deep Planning Network (PlaNet) is a purely model-based agent that learns environmental dynamics from images and selects actions through fast online planning in latent space. Using only pixel observations, the agent solves continuous rule tasks with contact dynamics, partial observability, and sparse rewards. This enables the models to solve more difficult tasks that were previously solved by planning with learned models (Hafner et al., 2019).

Quality diversity (QD) algorithms search for diversity of good solutions (Cully et al., 2015). Solutions of tasks to be optimized, usually encoded in high-dimensional parameter tuples, are stored in a low-dimensional feature space. This space is defined by a few often predefined properties, which may be based on behavioral, morphological, or other aspects of the solutions. The variety of solutions, all optimal in their own right, allows users to get an overview of how, for example, different optimized training plans can look like. It also provides an intuition of what options are available. QD has previously been used in motion planning in robotics, aerodynamic shape optimization, and urban planning, among others (Cully et al., 2015; Gaier et al., 2017a,b; Hagg et al., 2018, 2019; Urquhart and Hart, 2018).

In the field of optimization with multiple criteria to be optimized, iterative developments are visible. The optimization goal here is to always find trade-offs between criteria in a so-called Pareto front. It should be noted that new versions of such algorithms are available, such as the Non-dominated Sorting Genetic Algorithm in version 3 (Deb and Jain, 2013). There are novel developments in the optimization of more than three quality criteria, also called *many objective optimization* (Li et al., 2015). This field stands in contrast to QD, as it focuses on the functional diversity of solutions.

For optimization with computationally expensive criteria, statistical learning techniques are used in a Bayesian context. The idea is to evaluate only certain solutions and use these evaluations to train a simple statistical surrogate model, e. g. GP regression models. These surrogate models replace the expensive function in the majority of evaluations and are repeatedly updated with selected solutions. The solutions to be evaluated are selected based on the possible optimality predicted by the model. Combining the models ‘prediction and uncertainty results in an “optimistic” prediction, which has been shown to lead to efficient sampling (Jin, 2011; Shahriari et al., 2015). Bayesian optimization has also been used in the context of multi-criteria optimization (Emmerich et al., 2016) and QD (Gaier et al., 2017a; Hagg et al., 2020a). Moreover, generative models can also be applied to optimization and planning (Hagg et al., 2020b, 2021).

A very large field of research is reinforcement learning. Here, attempts are made to train AI models that try to solve a task themselves without much “supervision”. However, these methods are not unsupervised, as they still need some guidance during training. The methodology continues to attract significant attention, even in the non-specialist media, but is subject to many limitations that do not allow it to be applied in a human domain such as elite sports (Dulac-Arnold et al., 2021).

#### Interaction and Intervention

Athletes rely either on explicit indicators, such as the performance of the pursued goal, or on feedback, which can be intrinsic or augmented, to assess their performance. Intrinsic feedback is the information that arises from the athlete's perception of their own movements and position in space (i.e., proprioception), while augmented/extrinsic feedback is the information that comes from an external agent, such as the coach or video-based motion analysis (Mencarini et al., 2019).

Augmented feedback is important for learning and improving because it helps athletes categorize their internal sensations and better understand the mechanisms underlying their performance. This is the type of feedback that wearables can provide. Feedback can occur during or after the task. An example of an application of augmented feedback in the field of medicine is PARKIBIP, which uses IMUs to recognize gait phases of Parkinson's Disease people and encourages the patient to adjust the rhythm or step length with direct feedback using an app (Pasker et al., 2021). The decision whether to use concurrent or terminal feedback depends on the type of task required by the sport, as well as the content that the feedback is intended to express. In fact, feedback can express either knowledge of performance, if it refers to the quality of movements, or knowledge of results, if it refers to the goal/level achieved.

The detailed analysis in Mencarini et al. (2019) shows that research in human-machine interaction is in many ways still in its infancy. Interaction is seen from technical aspects of design, overlooking the impact of technology on user experience. They identify six directions of research on wearables for athletes: (1) while current research is dominated by wristwatch and concurrent feedback, research on “smart clothing,” for example, is still scarce; (2) research should go beyond proposing devices targeted at “average individual” athletes; (3) future research should also cover the complex constellation of cognitive, emotional and social aspects of the sports experience; (4) another intriguing research direction might be how technological artifacts can transform the sport experience by enabling radically new practices; (5) likewise, connecting wearable devices to the broader IoT ecosystem may open up new services and interaction modalities; (6) finally, more rigorous methodological approaches are needed for both user-needs analysis and technological artifact evaluation.

An important development is the integration of training – or motion sequences in virtual (VR) or augmented reality (AR). Nowadays, there are many approaches from different domains (Adhani and Rambli, 2012). The acceptance of VR and AR systems is investigated by Gradl et al. (2016) with 227 subjects. The results show that, about two-thirds of the participants are positive toward the use of VR and AR. Colley et al. (2015) describes a concept for using a head mounted display (HMD) while skiing and snowboarding. The wearer experiences an alternate VR visually through the HMD, while their other sensory inputs give the full sensation of skiing in the real world, creating a blended virtual/real experience. The prototype device has been evaluated in the real world. Feedback from athletes indicates that the level of immersion achieved is high. Furthermore, training applications are found in industry (Besbes et al., 2012), teaching (Dunleavy and Dede, 2014), sports through augmentation for compensating different abilities of players in ball sports (Sano et al., 2016), motion training on AR mirrors (Anderson et al., 2013), and haptic interfaces assisted by exoskeletons (Tsetserukou et al., 2010).

Generating simulated environments can also be partially done by generative models (Hsieh et al., 2019). In the aforementioned work, AI uses human sketches to help generate basketball simulations. This leads to a fast interaction between coach and player. The gap between simulation and reality, which certainly still exists, can be partially closed by simulation adaptation based on real data. Iterative and interactive methods are often used for this purpose (Chebotar et al., 2019; Xie et al., 2020).

In order to visualize, cluster, or otherwise analyze high-dimensional data, dimensionality reduction methods are often used. The data is often at or near a manifold of lower dimension than the original high-dimensional space. Dimensionality reduction transforms the data into a lower dimensional space and allows, for example, a clustering method to better distinguish clusters (Tomašev and Radovanović, 2016). One of the most commonly used methods is t-distributed Stochastic Neighborhood Embedding (t-SNE) (van der Maaten and Hinton, 2008).

## Status Quo of AI Usage in Elite Sports

### Methods

#### Literature Review

Although this is a narrative review, the aim of our literature review on the use of AI in elite sports was to follow the PRISMA 2020 checklist (Page et al., 2021) as far as possible. For the research we used the sport specific databases SportDiscus, SPONET and SPOLIT as well as the general databases Google Scholar, Journal Storage and SpringerLink (JSTOR). The first 50 entries of each database when using the following keywords, always in connection to “in elite sports” were examined: artificial intelligence, computer vision, sensors, wearables, power meter, text mining, speech recognition, machine learning, deep learning, ghosting, reinforcement learning, data mining, robotics, virtual reality, and visual analytics. With these key words we tried to cover the four steps of the SMPA loop as broadly as possible. Journals with a special interest in the AI, like the *Journal of Sports Analytics*, were evaluated as well. Because the pace of development of AI in sports within the last few years has been quite high, the focus of research has been on literature since 2010, if possible even since 2015. An important exclusion criterion was when a study was related to elite sport in a broader sense, but the focus was not on the sport itself. Examples for this would be Galily (2018) (sports journalism), Kakavas et al. (2020) (sports trauma prediction) or Nadikattu (2020) (sports business). A limitation regarding the selected methodology is certainly that completeness is not guaranteed. Rather, it was about finding out the distribution of applications of AI in elite sports across the four identified methodological categories *Image Processing, Signal Processing, Modeling & Planning* and *User Interaction*.

#### Expert Interviews

In order to discover how sports practice uses AI in training and competition, we interviewed nine experts from seven different countries (Australia, Austria, Canada, China, Germany, Russia, and Switzerland). Three of them were employed at the main national service institute for elite sports in these countries. The other six were employed at a sports science faculty at a university and thus have their focus in research. Nevertheless, they are still close to sports practice, since they often work directly with national teams in different sports and support them comprehensively. Each interviewed expert can be assigned to the intermediate area between sport and computer science. Appropriate written consent from participants to take part in the interviews and to publish the data has been obtained. The interviews were standardized using a guideline that was to be followed as much as possible in the interview. This guideline contained four different thematic blocks. First, the interviewees could give an overview of current or completed projects in the field of AI in elite sports. This section was used to obtain an impression of the advancement of AI in sports. To this end we asked for technologies used in the four identified methodical categories in sports specifically, which were mentioned in Section Literature Review. The interviewees provided us with specific examples for sports in which the technology is used. Based on these answers we were able to create a quantitative evaluation answering which categories of AI were applied to which sports. The second block was about conditions, which favor or hinder a successful project. The interviewees were asked about their experience in concluded projects, what to take into consideration to carry out a successful project, and what to avoid. We specifically asked, what special considerations should be made w.r.t. the different stakeholders of an AI project – the athlete/coach, the federations officials and the developer. In the third block, we asked the interviewees' opinion about the meaning of AI for theory building in sports science. This question was interesting in particular for the interviewees with a background in scientific research. Do they have to change their work now that technologies of AI are available? Can AI create new theories in sports science because of their possibilities to discover new patterns in data? Block four dealt with future developments regarding AI in elite sports. Here we asked in which field to expect the biggest steps in the next years.

### Results

#### Literature Review

**Figure 2:**
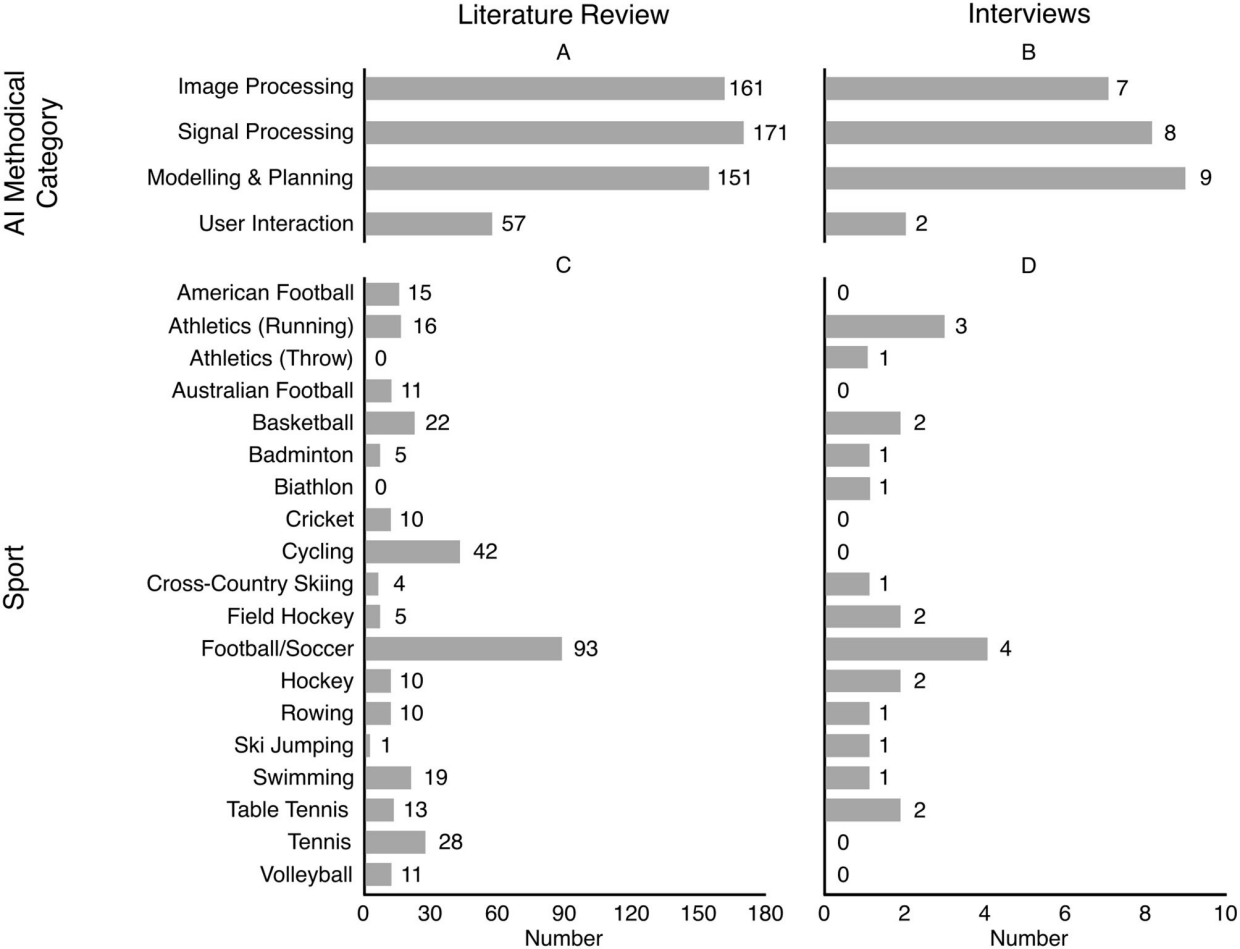
Number of found publications regarding **(A)** the methodical category, **(C)** specific sports; mentioned projects by the interviewees regarding **(B)** the methodical category, **(D)** specific sports.

Our Literature review found 540 publications regarding AI in elite sports. A number of publications do not treat one specific sport, but AI in elite sports in a general way. Here, 136 publications were found in total. In Figure 2C we show the number of found publications which treat one specific sport to gain an impression about the work regarding AI in elite sports and where it happened. The figure shows all sports with at least ten found publications with the criteria defined before. For reasons of consistency, sports with mentioned projects by the interviewees are also included, even if there are less than ten found publications. However, the aforementioned publications without the topic in one specific sport are excluded.

Figure 2A shows the distribution of publications grouped by the following categories: image processing, signal processing, modeling & planning, and user interaction. In total, there were 161 publications in the field of signal processing and 171 publications in the field of image processing. Thus, signal and image processing make up the largest part of the available literature. While image processing is mostly covered by the computer science community, signal processing is mostly part of the research of the sports science community. In endurance sports, like cycling, sensors – in the form of power meters – have played an important role for quite a time now. In running, power meters have become more popular within the last years, however not reaching the same importance compared to cycling (Aubry et al., 2018). Signal processing via sensors has the disadvantage that oftentimes, these devices are not allowed in competitions (i.e., 88). There is no such disadvantage with image processing, because cameras are usually positioned outside the area in which the competition takes place. Here, DL algorithms play an important role to analyze the recordings as exactly as possible. Cust et al. (2019) provide a wide overview of publications in the field of computer vision in different sports. It is noticeable that 39 of the 144 found publications can be assigned to football which makes up the largest proportion when we compare individual sports (Stensland et al., 2014; Manafifard et al., 2017; Linke et al., 2020). Other prime examples are publications regarding hockey (Tora et al., 2017), basketball (Ramanathan et al., 2016), and rugby (Kapela et al., 2015).

Within the last couple of years, the number of publications regarding the application of machine learning algorithms has starkly risen. We found 151 papers on this topic and regarding elite sports. Many papers are written on football and the big four American sports (American football, baseball, basketball, and hockey). The publications can be divided into prediction of results and technical-tactical analyses. While result prediction is interesting in particular for betting industries, technical-tactical analyses are of much more interest for coaches, athletes and sports scientists, as they produce tactical advice. In football, you can find a good overview of the use of ML at Herold et al. (2019) and Memmert and Raabe (2019). To highlight another publication in football, FIFA (2014) use DL algorithms to create ghost teams to analyze reactions of different tactical approaches. Examples of publications in the big four American sports are Tian et al. (2020) (basketball), Joash Fernandes et al. (2020) (American football), Kononenko (2001) (hockey) and Sidhu and Caffo (2014) (baseball).

Tools for mutual user interaction take up the smallest part of current literature (57 publications in total were found). The use of technologies like robotics and virtual reality is solely limited to training purposes, not for competitive use. Again, football takes up the largest proportion of found literature when analyzing one individual sport. Faure et al. (2020) provide us a wide overview of the use of VR. The authors evaluate 30 studies in different team sports regarding the use of VR in training. Other publications to be named are Covaci et al. (2015) (basketball), Vignais et al. (2015) (handball) and Correia et al. (2012) (rugby union). Visual analytics point to the challenge that, for example, findings of machine learning algorithms should not be misinterpreted because of unsuitable or insufficient input data. Visual analytics can analyze and clean data automatically, where humans can intervene in this process. Therefore, we include visual analytics to this methodical category. Keim et al. (2008) present to us a helpful overview of the use of visual analytics.

#### Expert Interviews

Our interviews largely confirmed the distribution of AI techniques in elite sports found by the literature review. Figure 2B shows the number of interviewees who mentioned AI projects with regard to the methodical categories already used above. Moreover, Figure 2D shows the distribution of mentioned projects regarding different sports.

The extensive collection of meaningful data is a main focus in the projects of most interviewees. Projects in image and signal processing were mentioned by seven and eight interviewees, respectively. In several sports this challenge is not solved sufficiently, according to coaches, athletes and analysts. Here, a shift from the more traditional way of data collection via sensors to a video-based data collection via computer vision can be observed. This is mainly because it was said that the collection of data is not allowed to affect the athlete during training or competition. One described example was that ski jumpers could react very sensitively if a sensor was attached to one of their skis, because it affects the air time during their jump. Therefore, data collection without wearables, via computer vision, is becoming increasingly important. This plays an important role in the acceptance of AI methods by athletes and coaches. Examples were mentioned by interviewees in which spatio-temporal data is collected using computer vision in sports like hockey or football. Nevertheless, data collection via wearables still plays a major role in most of the interviewees' everyday work. One large project of an expert uses inertial measurement units (IMUs) to collect large datasets about runners to create individual running profiles. This particular IMU device does not affect the runner in an obstructive way, according to the athletes, and is a sufficient technique for data collection. Finally, the challenge of data collection can only be viewed as (partially) solved in sports that enjoy large funding capabilities like football, American football or basketball. Underfunded sports, especially Olympic sports, which play an important role in the work of most interviewees, are faced with the complicated challenge of data collection without sufficient financial possibilities.

The interviewees were asked the following question: if a project within the identified methodical category of modeling & planning was conducted before? All the interviewees answered positively to this question. One example of such a project was the detection of tactical behavior in team sports, in particular in football and basketball. A restriction regarding a “full” AI (according to the SMPA loop) can be made here, because most experts mentioned, for example, that an ML algorithm cannot (or should not) make automatic action recommendations. This limitation is based on the fact that coaches need to stay in control of the decision process. Nevertheless, some projects were mentioned where automatic recommendations were made by the algorithm. One interviewee is working on a project, where an algorithm creates an automatic training plan for athletes in rowing, running and cycling. Convincing elite athletes to use such apps is a challenge not to be underestimated, as every small difference in training can have a significant impact on competitive performance. Similar to the challenge of data collection, projects mentioned in this field mostly cover sports with large funding capabilities.

The methodical category with the least mentioned projects was “user interaction”. Here, only two interviewees described concrete projects. One project involved the use of an exoskeleton to support injured athletes in their rehabilitation phase. The exoskeleton helps them to perform healthy movements when athletes are limited in their mobility. It is worth mentioning that projects of this methodical category can only be used in the training environment, not in competition.

As described in the method section, the second part of the empirical study dealt with general questions about how an AI project in elite sports can be successfully completed. Here, the interviewees mostly agreed that, a project should always be interdisciplinary (Vienni and Simini, 2018). At least one representative from all disciplines – computer science, sports science and sports practice – should be present. It is mandatory that the representative of sports science needs to understand the world of sports practice as well as have some experience with computer science. Thus, they should act as the link between the other two parties. Often it was referred to as a “translation challenge”. With a skilled sports scientist providing the link it is no longer necessary that the computer scientist has a deep understanding of the sport practice and vice versa. However, this overlap of skills is mostly welcomed. A general interest in sports as well as computer science is strongly recommended by nearly all interviewees. Another important element of a successful project that was mentioned is, that a project idea should have its origin in sports practice. This way it is more probable to succeed than the other way around, e.g., an AI research team using sports as an application.

The question regarding how AI can affect theory building in sports science was only relevant to some interviewees, because most of them work in facilities for direct application and support of sports. Here, the topic is not of particular interest. However, the interviewees of the research institutions confirmed the importance of AI in theory building. The possibilities of AI to find new, so far undiscovered, patterns in sports are highlighted here. On the other side, they express their concerns that the possibilities of classical methods are often overlooked. Often, specific research questions can be solved with classical methods, yet, AI methods are unnecessarily used. Classical methods, e.g., model based predictions, can return interpretable results and are usually less computationally expensive than novel AI methods. Again, an interdisciplinary approach should be considered to find the right methods.

About the future perspectives of AI in elite sports, every interviewed expert agreed that it will play a noticeably increasing role within the next years. More funding will probably be (or have to be) assigned to projects, whereas AI techniques, especially regarding data collection, probably will become cheaper. As already mentioned, nowadays, mostly underfunded sports still face the challenge of sufficient data collection. The unanimous opinion of the interviewees was that this challenge will and need to be addressed within the next couple of years. They agree that research focus will shift to the field of modeling & planning even more. However, opinions diverged regarding what specific techniques are expected to play a major role. To name one example, some highlighted the future possibilities of reinforcement learning algorithms in elite sports, like it was used in AlphaGo, while others were cautious here. Almost all interviewees expressed their concerns about having blind faith in the results of an AI solution. One should always question the results and keep a critical mind about it.

## Key Challenges for AI Usage in Elite Sports

In the following, we connect the results of our status quo analysis in sports and the opportunities that come with AI progress by enumerating six key challenges (Table 1). In order to bring the two perspectives together, we first mapped the identified four methodical categories for the use of AI in elite sports: (1) image processing, (2) signal processing, (3) modeling & planning, and (4) user interaction to the SMPA loop described in Section Introduction. Image and signal processing are techniques to sense actions of the environment (Figure 1, step 1). Whereas techniques of modeling & planning, like machine learning, cover the model building (Figure 1, step 2) as well as the planning and optimization (Figure 1, step 3). Techniques like virtual reality and wearables, which we assigned to the methodical category “user interaction”, cover the last part of the SMPA loop, acting on, or interacting with the environment (Figure 1, step 4).

**Table 1:** Challenges within elite sports with associated opportunities.

	**Challenge**	**Description**	**Opportunity**
1	Data collection	Sufficient data for underfunded sports is often unavailable.	Opportunities of transfer learning to use knowledge from well-funded fields.
2	Connecting AI and elite sports communities	Not enough AI researchers and developers find applications that originate in sports practice.	Networking between sports practitioners and AI scientist, e.g., through making sports data available to AI students and researchers. Vice versa AI methods can be made available to sports science students and researchers.
3	Keeping control in the hands of practitioners	Sports practitioners need to keep control of decisions.	Propose (interactive) plans to sports practitioners instead of automating decisions. AI methods should be seen as helpful support techniques. The feedback loop from sports practitioners back to the AI method is essential.
4	Explainability of AI results	Sports practitioners often express concerns about explainability of AI systems.	The subfield of “explainable AI” has a huge opportunity to provide a deeper connection between AI and sports, while learning how to deal with possibly novel problems. AI results should be visualized and communicated in ways appropriate to the field of sports science and sports practice.
5	Robust predictive models	AI needs predictive models that can be used with Small Data. In past years sports science mostly used models for data analysis and thus descriptive models that do not generalize well.	Most current models overfit due to the high number of parameters compared to the amount of data. Robust predictive models allow the use of models in the planning step of the SMPA loop.
6	Close SMPA loop	Most current applications of AI methods only implement part of the SMPA loop.	Since the SMPA loop often is not closed, the feedback to the AI system does not provide information about prediction and the effect of actions. Therefore, self-adaptation is not possible. Closing the loop usually involves athletes and trainers. Therefore, challenge 3 and 4 need to be considered.

### Challenge 1: Data Collection

In most sports, the collection of sufficient data still seems to be the main task. This satisfies the first part of the SMPA loop, the sensing of the (virtual) agent. To create a real AI, sufficient data must be collected in a way that ML methods can work with them properly. Generally, there are two options available to collect data: (1) via wearables and (2) via computer vision. Here, it first must be considered in which situations the one or the other are applicable at all. If wearables are not allowed in competition (like in football), it simply excludes the method of data collection and one must collect data via the other applicable method. In general, wearables have their advantage in the accuracy of the data, while computer vision's main advantage is that it does not affect the athletes at all.

Sometimes it is possible to combine camera data and data provided by wearables. For example, one could use position as well as fitness data of several players in team sports (as long as the rules do not exclude the use of wearables). With this opportunity for example, it would be possible to develop strategies that are more physically challenging to the opponent than to the players themselves. The fusion of such sensor networks offers a number of possible combinations and thus use cases that can provide an athletic advantage. Combining multiple sensor modalities can also lead to more accurate measurements (and thus more accurate predictive models) in elite sports.

Despite the amount of existing experience, there still seems to be a significant potential for improvements in the area of data collection in elite sports. However, one cannot deny that important steps were taken in the last years, and for a few sports the challenge of data collection can be viewed as mostly solved. Others can learn from these examples. It has to be taken into account that the data situation is not at all homogeneous in elite sports, nor can it be covered by a single method. On the one hand, certain large datasets are available, for example positional data in well-financed sports like football, American football, basketball, hockey or baseball. On the other hand, for most of the other (Olympic) sports no such large datasets exist. This restricts the use of (data-driven) AI methods in these sports dramatically, because DL methods need large datasets to be properly trained.

A promising opportunity lies in the use of transfer learning models. Models can be trained and validated based on large datasets from related sports and adjusted afterwards. This possibility was mentioned in a project by one interviewed expert. Transfer learning was used in field hockey while the model was originally trained on football data. In this specific case there is a clear potential to use transfer learning because of the undeniable similarity between these two sports (eleven vs. eleven players, similar sized playing field, etc.). It seems that there are more possibilities like this, for example in related racket sports like tennis, table-tennis and badminton.

### Challenge 2: Connecting AI and Elite Sports Communities

The opportunity to create a well-connected community of AI and sports practitioners should be strongly considered. Offering free and open data collections and methods would be met with a positive response within the AI community. The specific properties of sports data could then more easily feed into the development of robust AI methods. We recommend and encourage sports practitioners to share collected data.

During the planning phase of an AI project, the availability of modeling methods often stands or falls with the required funding and depends on licensing models. In this regard, we refer to the open source models and languages mentioned in Pouyanfar et al. (2018), which also support the integration of powerful graphics processing units (GPUs). The use of open source software is not only important for facilitating the funding of projects, but it should be noted that the majority of all machine learning experts work exclusively with open source software and are thus familiar with these tools. Access to computer scientists is thus more likely.

From the point of view of the AI community, methods could be made more accessible for sports science students and researchers. If there is a better methodological understanding in the sports community, it is more likely to not only start new projects regarding AI in elite sports, but also to push the projects toward success in the end.

Within the sports community, datasets could be shared among athletes to have specific problems solved with methods of AI that need greater data availability and variability. On the other hand, in terms of accuracy and generalization capabilities of AI systems with small datasets, it is also necessary to act methodically and to focus on such methods that do support smaller datasets.

### Challenge 3: Keeping Control in the Hands of Practitioners

Another important fact for the success of an AI project is the acceptance by the users. This can be increased by offering a variety of solutions – which can be computed using QD methods rather than single-objective optimization – if this variety can be well presented. Linking multiple criteria, such as performance and time efficiency, can be addressed by optimization methods. For example, solutions such as training plans can be presented as so called trade-offs, increasing the interaction between AI and trainer/athlete, and thus resulting in greater acceptance.

Data-efficient, online optimization can use surrogate-assisted methods to enable potentially novel interaction between AI and athlete. The interaction loop that emerges can potentially individualize models rapidly by having the AI make suggestions that are chosen and executed by the athlete. A feedback loop is created where training data and results can be fed back into the model. In this way, the athlete and AI work together to develop the best possible strategy. However, it must be noted here that the suggestions made by the AI are in the training-safe range. An important factor that should always be considered in elite sports is the robustness of optimization solutions. Just because a training plan based on certain data works well in a specific setup does not mean that the plan will continue to be of high quality in a changing environment in the future.

The acceptance of AI by athletes and coaches can also be increased if the methodology of AI is integrative, i.e., interactive and transparent. The interaction of coaches and athletes with the AI can make a significant difference here. The SMPA loop can be closed if the algorithm only suggests progressive and innovative solutions that stay within the usual risk framework. The importance of an ongoing interaction between coaches and athletes with the AI must be pointed out. This is important because of the unambiguous individuality of athletes in the context of elite sports. Sketching is also important as a craft tool in elite sports. By being able to automatically generate a simulation from the sketch, this importance can be magnified and also intervene in training.

Using an AI allows for novel solutions when optimizing plans and strategies. The exploration of new solutions and the exploitation of proven ones determine a parameterization dimension that allows coaches and athletes to systematically match opportunities and risks. As well as using deep generative models, methods like multimodal optimization and quality diversity, should be considered to replace single-objective optimization techniques. This can also help to fulfill the requirement of keeping the control in human hands while helping trainers and sports practitioners to discover novel plans and training strategies.

### Challenge 4: Explainability of AI Results

It is imperative that the explanatory power of models is incorporated into the application of elite sports. This is the only way to convince participants, both coaches and athletes, that model predictions are useful and will remain useful in the future and in unexpected situations. The AI subfield of “explainable AI” specifically targets this challenge.

In order to increase the transparency and understanding and thus the acceptance of AI systems, the visualization of results must always be designed to be as informative but also as effective as possible. Here, among other things, dimensionality reduction methods can be helpful when complex data or optimization results need to be communicated. The use of generative models can be used for visualizing high dimensional data and optimizing results. This allows users, coaches and athletes alike, to develop a better understanding of the data and results.

Virtual and augmented reality allow feedback between AI and athletes under controllable and changeable environmental conditions. Augmented reality can increase the acceptance of feedback systems in elite sports. There can be interaction and adaptation based on real data when using simulated environments.

Validation, but also ongoing reassessment of system performance, should also be a priority. Learning models do just that: learn and adapt. Thus, system performance that adapts must also be re-evaluated. Adaptations have to be transparent and explainable. The AI system has to be able to provide some evidence for the change in a human-readable form.

For prediction, the use of statistical, “white box” models, such as GP regression models, should always be explored as an alternative to black box data-driven AI methods. There are still domains where these more classical methods deliver faster, better and more explainable results than AI methods. Statistical models are well understood outside the field of machine learning. Such models provide confidence intervals over their predictions, which can support and put into perspective the validity of models. Especially in borderline cases, model confidence for risk avoidance should always be considered. GP regression models can also be used for sports with larger data sets and should therefore be considered as a statistical and more robust alternative to other methods, like DL. In the case of large amounts of unqualified data, unsupervised learning methods should be taken into account in research projects. They can be used in the field of sports data as a partial substitute for model training.

### Challenge 5: Robust Predictive Models

Most current models are used for analyzing obtained data rather than for prediction. Often they include a large number of interpretable parameters – if derived from first principles – or other free parameters to allow for a good data fit. Unfortunately, this often leads to overfitting the small data available which results in poor prediction performance. Since in AI, the major use of models lies in prediction, this fact is a major cause preventing the use of ML models in AI systems.

Robust predictive models can be used in the planning step of the SMPA loop, while taking into account measurement noise and dynamic environments, which includes changes in athletes' own physique. As already done for analysis models in injury risk assessment, predictive models should be developed to put more emphasis on robust prediction. In such cases where data from the elite sports domain is noisy or erroneous, robust models can still be trained even based on limited datasets. This can be done by artificially generating data with generative models and by making use of transfer learning, which allows us to use structures learned from larger data sets. Experience from the medical field, especially for small data sets, should be transferred onto the sports domain.

By and large we can decide which method is most effective and robust, only if both classical and novel ML methods are considered and validated. The robustness of systems should be tested during their development by examining when their accuracy and reliability break down. This includes testing in the real world and not only on static data sets.

### Challenge 6: Close SMPA Loop

Most current applications of AI methods in sports only implement part of the SMPA loop (Figure 1). As mentioned in challenge 5, models are often only used for analytical purposes. Since the SMPA loop is not closed, no feedback is provided to the AI system. Therefore, no information about the quality of prediction and the effect of planned actions is available to the system. As a consequence, self-adaptation – probably the main characteristic of AI systems – is not possible.

To close the AI loop Challenge 1 and 6 need to be solved as well. However, closing the loop also involves athletes and trainers, therefore, Challenge 3 and 4 need to be considered. Moreover, interaction between human and machine plays a central role in this challenge. By offering athletes and trainers a diverse and explainable set of possible plans or instructions, we can provide feedback while keeping the human in control. This will increase the acceptance by the users. Additionally, it will provide the opportunity to modify the next iteration of the SMPA loop by including the feedback of the real world application using an AI's suggestion. The reintegration of (multimodal) feedback systems into the SMPA loop, which provides the AI system with opportunities for self-adaption, is a necessary part to reach full AI integration into assistive systems.

## Summary and Conclusion

**Figure 3:**
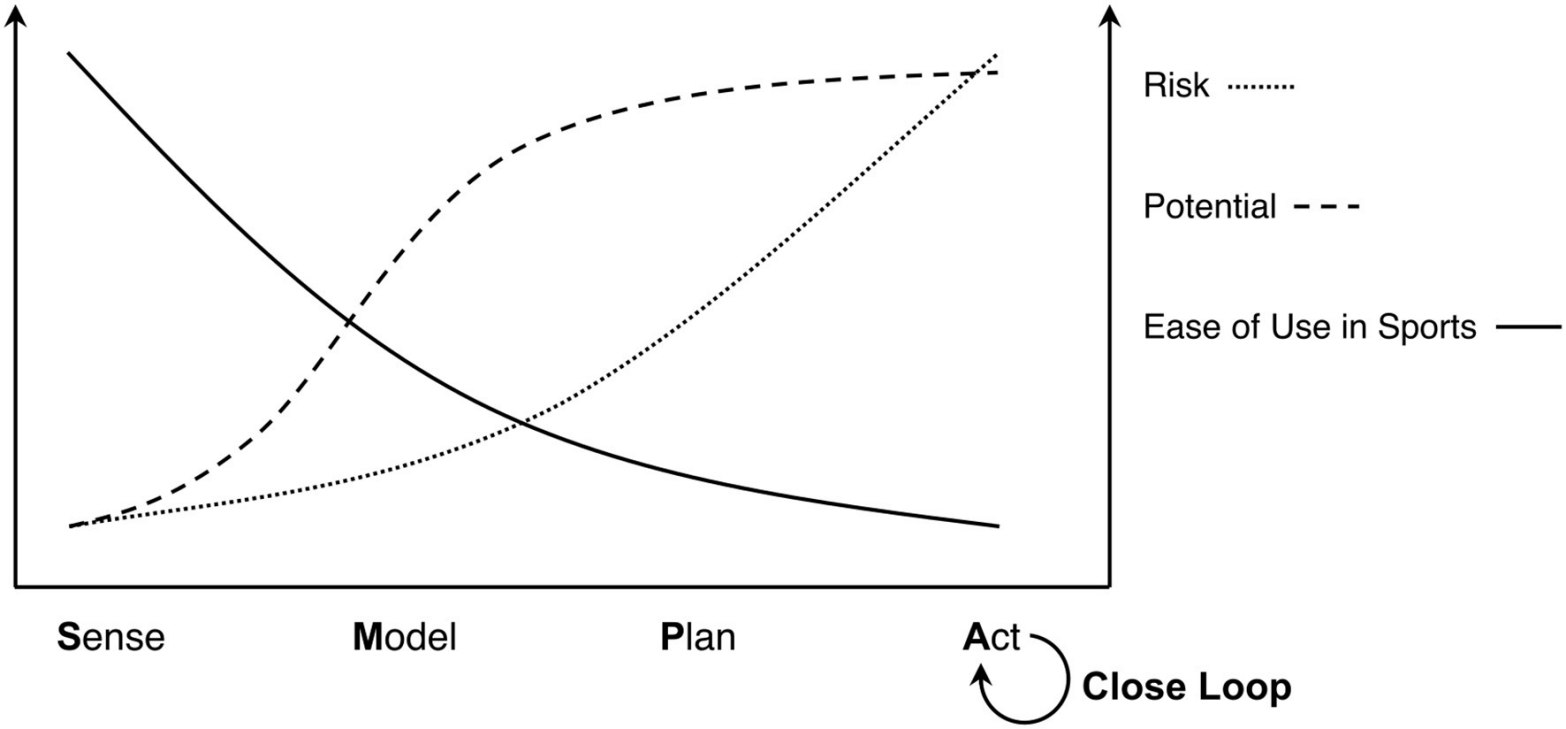
Risk, potential and ease of use in sports of the four steps in SMPA loop.

Based on the overview of success stories of AI outside the sports domain (chapter 2) and the status quo of AI in sport (chapter 3) we identified six challenges regarding the use of AI in elite sports, namely – data collection, connecting AI and elite sports, keeping control in the hands of practitioners, explainability, robust predictive models and finally closing the SMPA loop. Closing this loop holds the biggest potential in creating a performance improving environment for athletes, but is obviously the ultimate challenge in building AI systems since, each of the subsystems has to be implemented. This means that the risk of a project failure when implementing a closed AI loop in an elite sports environment seems to be quite high due to technical difficulties or acceptance problems. On the other extreme, sensors for data collection and analytic models are quite easy to use and elite sports can directly profit from current developments in AI. Currently the return on investment in this area is high (with low risk due to technical problems or acceptance problems) but the potential in creating a performance improving environment is rather low. Figure 3 tries to visualize the relation that the potential and risk of AI developments are increasing with every step in the SMPA loop, whereas developments especially in the planning and acting step are quite difficult to implement in sports.

We think that within the next few years the best balance between risk and potential probably lies in the use of ML methods for predictive modeling, as the risk is not too high. This is particularly important when dealing with underfunded sports. In this work, we pointed out various options that can be used to apply ML in sports. In addition, the majority of experts that were interviewed in this study concluded that developments of AI in sports in the near future will mostly be in the field of ML.

In the Sense step of the SMPA loop, many obvious opportunities exist and have been used for state of the art research. ML has been applied in sports, but most results are not convincing as of yet. We do recognize its potential, especially with methods like transfer learning that allow the application of ML methods on smaller data sets. Planning has not been focused by the ML and sports research communities, but ultimately it is important to be able to close the SMPA loop in order to provide a self-adaptive AI system to assist athletes and trainers.

## Data Availability Statement

The raw data supporting the conclusions of this article will be made available by the authors, without undue reservation.

## Ethics Statement

Ethical review and approval was not required for the study on human participants in accordance with the local legislation and institutional requirements. The patients/participants provided their written informed consent to participate in this study.

## Author Contributions

FH, AH, AA, and DL contributed to conception, design of study, and wrote sections of the manuscript. FH executed the interviews. FH and AH wrote the first draft of the manuscript. All authors contributed to manuscript revision, read, and approved the submitted version.

## Funding

This study was supported by the German Federal Institute of Sport Science (BISp, www.bisp.de), grant number 080602/19-20. This institution had no role in study design, data collection and analysis, decision to publish, or preparation of the manuscript.

## Conflict of Interest

The authors declare that the research was conducted in the absence of any commercial or financial relationships that could be construed as a potential conflict of interest.

## Publisher's Note

All claims expressed in this article are solely those of the authors and do not necessarily represent those of their affiliated organizations, or those of the publisher, the editors and the reviewers. Any product that may be evaluated in this article, or claim that may be made by its manufacturer, is not guaranteed or endorsed by the publisher.
