# Supplementary material for: Artificial Intelligence in Elite Sports—A Narrative Review of Success Stories and Challenges
Source: Front Sports Act Living. 2022 Jul 11;4:861466. doi: 10.3389/fspor.2022.861466 (PMC9309390; doi:10.3389/fspor.2022.861466)
Supplement: Supplementary file 1 [file Data_Sheet_1.pdf]

Guideline expert interview

Entrance:

- Thank you for your time to talk with us about the interesting topic of artificial intelligence in competitive sports.
- Like we said via E-Mail we work on a project regarding AI in Top Level Sports and wanted to ask national and international experts about their opinion regarding this topic. We want to get an overview about the level of AI in Top Level Sports.
- For how long do you work in the field of Sports Analytics respectively AI in Sports and what was your way into this field?

Main part:

- We chose a very broad definition of AI for the project. So, I would name some different AI technologies now and I would ask you to tell me if these technologies
  - Are in use at this time. If yes, in which Sports/Projects?
  - What is the goal? Which parameters are raised? What's the advantage compared with conventional approaches?
  - are planned to use in projects until 2024?
  - Could be used in a long term (until 2030)?
    - Image recognition/Computer Vision
    - Sensors
    - Speech recognition/Speech processing
    - Text Mining
    - Machine Learning/Deep Learning
    - Reinforcement Learning
    - Robotics
- Are there any other AI technologies I didn't mention?
- Do you have experience that AI creates recommended actions automatically for training or competition purposes? Could this be possible short or long term (until 2030)?
- When you look back to your projects. Can you name conditions which favor a successful project execution or which hinder a successful project execution with regard to the stakeholder:
  - Developer
  - Tool User (Coach…)
  - Officials
- We suppose that it is extremely important that the interdisciplinary (Sports vs. Informatics, Theory vs. Practice) is given. What is your experience here? Do you have recommendations what to do for a successful project?
- Is AI in use for sports theory/sports modeling? For example, does AI create new tactical concepts? If yes, can you give us an example? And how large is the importance?
- I come to the end now: May you please give me an outlook how important AI in top level sports will be in the future? How will it develop? And what are important requirements for a good development?
- Do you want to add anything?

End:

- You will receive the results of our project when we finish it.
- Thank you very much for your time.
